# Occurrence and Identification of Pathogenic *Vibrio* Contaminants in Common Seafood Available in a Chinese Traditional Market in Qingdao, Shandong Province

**DOI:** 10.3389/fmicb.2020.01488

**Published:** 2020-06-30

**Authors:** Xinjie Song, Jinlin Zang, Weisen Yu, Xuexiang Shi, Yongning Wu

**Affiliations:** ^1^School of Biological and Chemical Engineering, Zhejiang University of Science and Technology, Hangzhou, China; ^2^Qingdao Municipal Hospital, Qingdao, China; ^3^Department of Nutrition and Food Hygiene, Qingdao Center for Disease Control and Prevention, Qingdao Institute of Preventive Medicine, Qingdao, China; ^4^NHC Key Laboratory of Food Safety Risk Assessment, Chinese Academy of Medical Sciences Research Unit (2019RU014), China National Center for Food Safety Risk Assessment, Beijing, China

**Keywords:** Foodborne disease surveillance, *Vibrio alginolyticus*, *V. parahaemolyticus*, *V. vulgaris*, sea snails

## Abstract

The investigation of the causative agents for foodborne diseases and subsequent development of preventive steps to control the outbreak and related economic loss is the basic goal and priority of a rational food safety program. The entero-pathogenic *Vibrio* spp., which are Gram negative bacteria inhabiting estuarine ecosystems, are the major cause of foodborne illness associated with the consumption of raw or undercooked contaminated seafood or shellfish. To survey the *Vibrio* contamination in sea snails (*Neptunea cumingi Crosse* and *Busycon canaliculatu*), a total of 20 samples were collected from traditional market, at Qingdao city in Shandong province, China and analyzed for *Vibrio* species contamination. Presumptive-positive colonies grown on a specific *Vibrio* agar-based medium were picked and identified by the VITEK^TM^. *Vibrio alginolyticus*, *V. parahaemolyticus*, and *V. vulgaris* were isolated and identified in 11, 8, and 2 seafood samples, respectively. Among the 8 isolates of *V. parahaemolyticus*. The *V. parahaemolyticus* isolates were further tested for the *tdh*, *trh*, and *tlh* virulence factors. All the *V. parahaemolyticus* isolates were *tlh*-positive, however, all of them were *tdh*-negative. Interestingly 2 *V. parahaemolyticus* isolates were positive for *trh* virulence factor. These results indicated that there is a high incidence of *V. alginolyticus* and *V. parahaemolyticus* in sea snails. Therefore, food safety regulations for fishery auction markets should be established to control these species in addition to other *Vibrio* pathogenic contaminants. Our study provides the first evidence for the prevalence of *Vibrio* spp. in sea snail samples from traditional market in the Qingdao province of China.

## Introduction

Sea snail is a nutritious food that contains various desirable nutritional components of a healthy diet. Nevertheless, there are many health risks associated with the careless consumption of seafood products (Zhang et al., 2018). One of the major risks, seen in a few Asian countries, including China, Korea, and Japan, involves the consumption of raw or undercooked seafood that may be naturally contaminated by foodborne pathogens present in the marine environment (Weissfeld, 2014). The risk of marine contamination can be increased if the food is mishandled during production and processing, allowing microorganisms to multiply exponentially in favorable environments (Amagliani et al., 2012). *Vibrio* spp. are Gram negative, facultatively anaerobic, rod shaped, non-spore-forming bacteria that are ubiquitously and naturally present in estuarine waters throughout the world’s aquatic environments and are especially resistant to high salt concentrations (Su and Liu, 2007). *Vibrio* was first identified as a foodborne pathogen in Japan in the 1950s (Fujino et al., 1953; Letchumanan et al., 2019). By the early 1970s, *Vibrio parahaemolyticus* was recognized as a cause of diarrheal disease worldwide, most commonly in Asia and the United States. *Vibrio parahaemolyticus* associated with sea foods is one of the leading agent for food borne illness in China and across the world (Chen et al., 2017; Yang et al., 2017). Although cooking destroys this organism, sea snails are sometime eaten raw and will lead to infections associated with *V. parahaemolyticus*. Therefore, factors contributing to *Vibrio* contamination, including improperly handled seafood products, could pose a risk of exposure to these infectious agents that are transmissible to humans. Several species of the genus *Vibrio* are associated with foodborne spoilage and contamination of seafood products (Heng et al., 2017; Lee et al., 2018). The route of *Vibrio* entry into the biliary system remains unknown, and it was assumed to be via cutaneous lesions or due to gastrointestinal translocation upon consumption of contaminated seafood, as in other *Vibrio* infections (Liu et al., 2011). A few other species are more hazardous to humans, including *V. parahaemolyticus* and *Vibrio cholerae*, which are known to cause severe intestinal diseases (Kass and Riemann, 2006). The majority of *V. parahaemolyticus* isolates are avirulent, but they are still the leading cause of gastroenteritis linked to seafood consumption in several countries, including the United States (Iwamoto et al., 2010). Some *Vibrio* spp. pose a significant health risk to the immunocompromised elderly people and children. *Vibrio* may enter human hosts via wounds or direct consumption of raw seafood, including shellfish (primarily oysters), and frequently lead to septicemia and, ultimately, death in susceptible individuals (Harwood et al., 2004).

Researchers have shown a significant association between rising seawater temperatures and an increase in the number of *Vibrio* infections, suggesting that global warming might also be a factor affecting the increasing incidents of *Vibrio* contamination in seafood (Huehn et al., 2014). Over the past few years, *V. parahaemolyticus* strains have often been isolated from fish, shrimp, and other types of seafood. Additionally, Cho et al. (2016) and Mala et al. (2016) isolated *V. parahaemolyticus* in ready-to-eat food. In the Zhejiang province of China alone, there were 71 outbreaks caused by *V. parahaemolyticus*, resulting in 933 illnesses and 117 hospitalizations from 2010 to 2014. Recently, a multistate outbreak of gastrointestinal illnesses linked to oysters imported from Mexico occurred in June 2019; 16 people became severely ill, and almost all patients had *Vibrio* infections, especially those involving *V. parahaemolyticus* and *Vibrio albensis* (CDC, 2019).

Reports of outbreaks causing life threatening infections with *Vibrio* species associated with the consumption of a variety of seafoods are increasing. Therefore, to understand the risk of acquiring *Vibrio* infections through the consumption of traditional market-based seafood, it is important to have data on the prevalence of the virulent strains in traditional market-based seafood. This study aimed to examine the distribution of *Vibrio* species in sea snail (*Neptunea cumingi Crosse* and *Busycon canaliculatu*), Chinese traditional market-based seafood in the Qingdao province of China and determine the prevalence of the virulence genes of thermostable direct hemolysin (*tdh*), thermostable direct hemolysin-related hemolysin (*trh*), and thermolabile hemolysin (*tlh*) in *V. parahaemolyticus* isolates for food safety purposes.

## Materials and Methods

### Sample Collection

**FIGURE 1:**
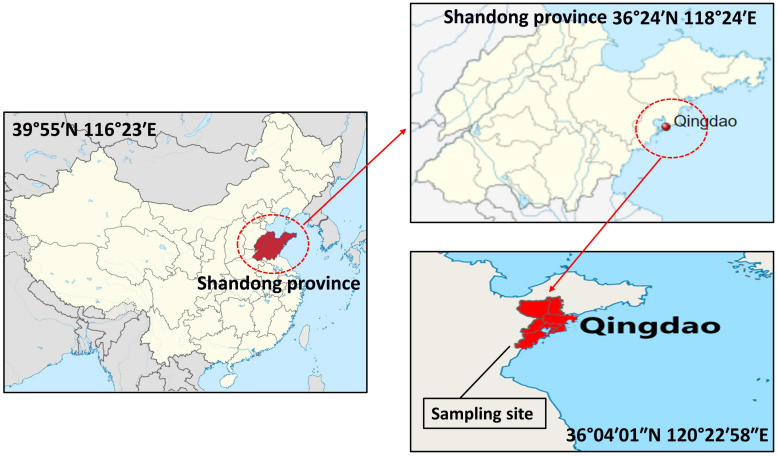
Map of samples (sea snail) collection site in Qingdao city (36°04′01″N 120°22′58″E) of Shandong province, China.

In total, 20 samples of sea snail were randomly collected from four traditional markets in the Chinese city of Qingdao (36°04′01″N 120°22′58″E) during March–September, 2018 (Figure 1). Approximately 1 kg of each sample was collected. All samples were packed in sterile plastic zipper bags, stored in an airtight box containing crushed ice, and transferred to the laboratory in 4 h for further processing.

### Sample Processing

All samples were thawed and pre-incubated in buffered peptone water for 16–18 h. Thereafter the samples were serially diluted, cultured and the bacteria were counted according to standard microbiological methods. Briefly, 25 g of each seafood sample was transferred into a sterile stomacher bag under aseptic conditions and blended with 225 mL of sterile buffered peptone water using a homogenizer set at 200 rpm for 15–20 min. Further, serial dilutions were made using sterile 0.1% peptone water to allow for culturing and bacterial enumeration.

### Microbiological Analysis

*Vibrio* spp. were isolated and enumerated from all samples. The bacterial counts were determined using *Vibrio* spp. selective thiosulfate citrate bile salt (TCBS) agar plates. TCBS agar plates were inoculated by spreading 0.1 mL of the serial dilutions of samples and incubated at 37°C for 48 h. TCBS plates were examined for the presence of either yellow, round, 2–3 mm diameter colonies (suspect: *V. alginolyticus*) or green, round, 2–3 mm diameter colonies (suspect: *V. parahaemolyticus*). Each isolated suspected *Vibrio* colony was streaked onto another TCBS agar plate and incubated overnight at 37°C to obtain pure colonies.

### Biochemical Characteristic Analysis

VITEK 2^TM^ (BioMérieux, Marcy-l’Étoile, France) automated system was used according to the manufacturer’s instructions to identify the *Vibrio* spp. The method is a combination of photoelectric technology, computer technology, and bacterial octet digital identification technology. VITEK is frequently used for identification owing to its simplicity, accuracy and prompt outcome (Funke et al., 1998). In VITEK 2^TM^ system, each test card contained 64 wells to carry out biochemical reactions (Funke et al., 1998). Each group was divided into three terms, and each positive reaction in the group was evaluated as 1, 2, 4, and then the values of each group were calculated. Based on the 64 biochemical reactions, biological identification numbers were obtained. With the help of the computer, the test cards were read after every 15 min and the dynamic reaction changes could be observed via optical scanning of the substrate of each reaction hole.

The specific operation procedure was as follows. The isolated bacteria were streaked on TCBS plates and incubated for 24 h at 35°C. A homogeneous suspension was prepared with an optical density (600 nm) between 0.55 and 0.63, measured with a DensiCHEK^TM^ VITEK Caliper. Sixty-four biochemical tests were performed using the gram-negative bacteria identification VITEK cards. The VITEK biochemical tests for *Vibrio parahaemolyticus* (yellow color colony in TCBS medium) are described positive for indole, nitrate reduction, citrate utilization, and growth at 7% NaCl, while negative for urease, arginine, sucrose fermentation, swarming, and growth at 10% NaCl. Whereas *Vibrio alginolyticus* (appeared a*s* green color colony in TCBS medium) are positive for indole, Voges–Proskauer (VP), nitrate reduction, citrate utilization, sucrose fermentation, swarming, and growth at 7 and 10% NaCl, while negative for urease, arginine.

### Virulence Gene Detection

The virulence genes, namely *tlh* (450 bp) (Gutierrez West et al., 2013), *tdh* (251 bp) (Tada et al., 1992), and *trh* (250 bp) (Tada et al., 1992), were tested by polymerase chain reaction (PCR) amplification. The sequences of primers were as follows:

tlh-F: 5′-AAAGCGGATTATGCAGAAGCACTG-3′; tlh-R: 5′-GCTACTTTCTAGCATTTTCTCTGC-3′; tdh-F: 5′-CCACTACCACTC TCATATGC-3′;
tdh-R: 5′-GGTACTAAATGGCTGACATC-3′; trh-F: 5′-GGCTCAAAATGGTTAAGCG-3′; trh-R: 5′-CATTTCCGCTCTCATATGC-3′.

The PCR was carried out in a final volume of 20 μL containing 1 × PCR buffer (containing MgCl_2_) (Takara, Dalian, China), 0.5 mM dNTPs (Takara), 0.25 μM forward primer and reverse primer (Songon, Shanghai, China), 1.0 U Taq polymerase (Takara), and 50 ng genomic DNA. The PCR amplification was conducted under the following conditions: initial denaturation at 94°C for 5 min, followed by 30 cycles of denaturation at 94°C for 1 min, annealing at 55°C for 45 s, and extension at 72°C for 45 s, and an additional 7-min extension at 72°C. The amplicons were analyzed by 1.2% agarose gel electrophoresis and photographed in the gel imaging system (Vilber Lourmat, France). The amplicons were gel purified by TIANgel Midi Purification Kit (Tiangen) and send to Sangon Biotech Co., Ltd. for sequencing to confirm the identities. The sequence data were compared with those in the NCBI nucleotide sequence database by means of Basic Local Alignment Search Tool.

## Results and Discussion

Total 35 *Vibrio* spp. were isolated from the tested 20 seafood samples using selective (TCBS) agar medium. All the isolates were tested for three major *Vibrio* species: *V. parahaemolyticus*, *V. alginolyticus*, and *V. vulgaris* using VITEK test kits, and tested for biochemical activity, serotype, and the presence of known virulence markers (*tlh*, *tdh*, and *trh* genes). The *tdh* and *trh* genes are strongly correlated with the virulence of pathogenic *V. parahaemolyticus* (Gutierrez West et al., 2013; Raghunath, 2015). To our knowledge, very few studies have characterized such a diverse panel of *V. parahaemolyticus* isolates in seafood samples from traditional markets at this level. Table 1 shows detailed information on the *Vibrio* species identified in all samples. The reliability and reproducibility of the VITEK system for *Vibrio* strain identification was tested every other day. Out of the 20 seafood samples, *V. alginolyticus* was identified in 55% (*n* = 11), while *V. parahaemolyticus* was identified in 40% (*n* = 8) samples. Interestingly, *V. parahaemolyticus* is highly co-occurred with *V. alginolyticus* (8/11). Besides these two *Vibrio* spp., *V. vulgaris* was also present in 10% samples (*n* = 2), and it always co-occurred with *V. alginolyticus* and *V. parahaemolyticus* (Table 1). These results indicate that there is a high incidence of *V. alginolyticus* and *V. parahaemolyticus* in seafood distribution channels. The *V. parahaemolyticus* isolates were further tested for the *tdh*, *trh*, and *tlh* virulence factors. Of these, *tlh* is suggested to be an important molecular marker for the identification of *V. parahaemolyticus* (Gutierrez West et al., 2013). Results showed that all the VITEK-identified *V. parahaemolyticus* isolates (*n* = 8) were positive for *tlh*, thus confirming the success of the VITEK identification method. Results are good agreement with the previous published reports showing the presence of *tlh* positive *V. parahaemolyticus* in many sea food samples (Letchumanan et al., 2014). Consistent with this Terzi Gulel and Martinez-Urtaza (2016) identified and confirmed many strains of *V. parahaemolyticus* using *tlh* virulence marker. We further noticed that among all the identified *V. parahaemolyticus* isolates, only 25.0% (*n* = 2) were positive for *trh*. Moreover, all the identified *V. parahaemolyticus* isolates were *tdh*-negative. These results are consistent with the findings of Jones et al. (2012) who obtained *tdh*-negative and *trh*-positive isolates of *Vibrio* species from Canada, Maine, and Washington during the early summer, indicating a potential preferential distribution of these strains in northern areas. A study carried out by Tran et al. (2018) on 385 sea food samples demonstrated the presence of *tdh* and *trh* positive *V. parahaemolyticus* strains in 25 samples. Out of theses 25 samples, only 2 samples have *V. parahaemolyticus* strains with both *tdh* and *trh* gene and rest 23 samples have *V. parahaemolyticus* strains either with *tdh* and *trh* virulence gene. In another similar study done by Yang et al. (2017) examined the prevalence of *V. parahaemolyticus* in various oyster, fish and shrimp samples collected form the South China domestic market and revealed 8.16 and 12.24% *tdh* and *trh* positive isolates, respectively. The difference in the distribution and evolution of *tdh* and *trh* in *V. parahaemolyticus* in different samples are highly associated with geographical location, sample source and environmental conditions (Raghunath, 2015). This was also reflected in a previous report that *trh*-positive *V. parahaemolyticus* strains constitute a higher proportion of the total *V. parahaemolyticus* population in the Mid-Atlantic region than in other areas during the summer (DePaola et al., 2010).

**TABLE 1:** Positive samples of *Vibrio* species in sea snail available in a traditional market in Qingdao city, Shandong province, China.

Positive sea snail samples	TCCB Agar plate*	VITEK Identification	Presence of virulence gene		
			tlh	tdh	trh
1	+ve for *Vibrio* spp.	*V. alginolyticus*	−ve	−ve	−ve
2	+ve for *Vibrio* spp.	*V. alginolyticus*	−ve	−ve	−ve
3	+ve for *Vibrio* spp.	*V. alginolyticus*	−ve	−ve	−ve
		*V. parahaemolyticus*	**+ve**	−ve	**+ve**
4	+ve for *Vibrio* spp.	*V. alginolyticus*	−ve	−ve	−ve
		*V. parahaemolyticus*	**+ve**	−ve	−ve
5	+ve for *Vibrio* spp.	*V. alginolyticus*	−ve	−ve	−ve
		*V. parahaemolyticus*	**+ve**	*-ve*	*-ve*
6	+ve for *Vibrio* spp.	*V. alginolyticus*	−ve	−ve	−ve
7	+ve for *Vibrio* spp.	*V. alginolyticus*	−ve	−ve	−ve
		*V. parahaemolyticus*	**+ve**	−ve	−ve
8	+ve for *Vibrio* spp.	*V. alginolyticus*	−ve	−ve	−ve
		*V. parahaemolyticus*	**+ve**	−ve	−ve
		*V. vulgaris*	−ve	−ve	−ve
9	+ve for *Vibrio* spp.	*V. alginolyticus*	−ve	−ve	−ve
		*V. parahaemolyticus*	**+ve**	−ve	−ve
		*V. vulgaris*			
10	+ve for *Vibrio* spp.	*V. alginolyticus*	−ve	−ve	−ve
		*V. parahaemolyticus*	**+ve**	−ve	−ve
11	+ve for *Vibrio* spp.	*V. alginolyticus*	−ve	−ve	−ve
		*V. parahaemolyticus*	**+ve**	−ve	**+ve**

*TCBS, thiosulfate citrate bile salt (Vibrio spp. Selective medium); −ve: negative; +ve: positive. Bold value is highlight of the positive result.

Similar to our study, *V. alginolyticus* was also found to be the most frequently isolated species in two culture-based studies from Italy and Malaysia (Elhadi et al., 2004). In 2011, an interesting study on the prevalence of *V. parahaemolyticus* in retail oysters sold at a local market in São Paulo State (Brazil) reported that 100% of the samples was contaminated with *V. parahaemolyticus*. In addition, none of the 1,943 tested isolates in that study harbored *tdh* and/or *trh* gene(s) (Sobrinho et al., 2011). Our data provides the first information on exposure to *Vibrio* spp. in traditional market-based seafood in China. However, it must be recognized that using only culture-based methods to detect *Vibrio* spp. could be a limitation. In our study, the VITEK along with *tdh*, *trh*, and *tlh* virulence factors identification system was used to identify the presence of potentially pathogenic *Vibrio* spp. in traditional market-based seafood.

## Conclusion

This study identified several *Vibrio* species in sea snail samples locally available in the traditional Chinese market of Qingdao city, Shandong province, China through culturing on selective media and VITEK biochemical techniques. *V. alginolyticus* was found in 55% (*n* = 11) of selected sea snails while *V. parahaemolyticus* was found in 40% (*n* = 8) of the samples. Besides, *V. vulgaris* was also present in 10% samples (*n* = 2), and it always co-occurred with *V. alginolyticus* and *V. parahaemolyticus* in samples. All *V. parahaemolyticus* isolates were positive for *tlh* virulence factor. In addition 2 *V. parahaemolyticus* isolates found positive for *trh* virulence factor. Eating raw seafood in Asian countries, including China, Korea, and Japan is the main cause of emerging gastrointestinal diseases. Therefore, based on the results obtained in this study, further efforts are needed for rapid pathogen identification and for controlling *Vibrio* contamination in sea snails. Additionally, meta-analyses or systematic studies on the worldwide prevalence of antimicrobial-resistant *V. parahaemolyticus* in seafood are needed. Furthermore, to minimize the risk of *V. parahaemolyticus* infection and ensure seafood safety, consultation and collaboration between researchers and governments are urgently needed. We further suggest avoiding the consumption the raw sea snails owing to their high chance of contamination with *Vibrio* species.

## Data Availability Statement

The raw data supporting the conclusions of this article will be made available by the authors, without undue reservation, to any qualified researcher.

## Author Contributions

XSo wrote the manuscript. XSo, JZ, WY, and YW performed the experiments and analyzed the data. XSh evaluated the drafts of manuscript and approved the manuscript for submission. All authors contributed to the article and approved the submitted version.

## Conflict of Interest

The authors declare that the research was conducted in the absence of any commercial or financial relationships that could be construed as a potential conflict of interest.
